# Osmotically Activated Anion Current of Phycomyces Blakesleeanus—Filamentous Fungi Counterpart to Vertebrate Volume Regulated Anion Current

**DOI:** 10.3390/jof9060637

**Published:** 2023-05-31

**Authors:** Katarina S. Stevanović, Bogdana Čepkenović, Strahinja Križak, Miroslav Ž. Živić, Nataša V. Todorović

**Affiliations:** 1 Faculty of Biology, Institute of Physiology and Biochemistry, University of Belgrade, Studentski Trg 16, 11158 Belgrade, Serbia; katarina.stevanovic@bio.bg.ac.rs (K.S.S.); bogdanacepkenovic@outlook.com (B.Č.); mzivic@bio.bg.ac.rs (M.Ž.Ž.); 2 Institute of Multidisciplinary Research, University of Belgrade, Kneza Višeslava 1, 11030 Belgrade, Serbia; strahinjakrizak@yahoo.com; 3 Institute of Biological Research “Siniša Stanković“, National Institute of the Republic of Serbia, University of Belgrade, Bulevar Despota Stefana 142, 11000 Belgrade, Serbia

**Keywords:** osmotic stress, ATP, nitrate, glutamate, patch-clamp, single channel current

## Abstract

Studies of ion currents in filamentous fungi are a prerequisite for forming a complete understanding of their physiology. Cytoplasmic droplets (CDs), obtained from sporangiophores of Phycomyces blakesleeanus, are a model system that enables the characterization of ion currents in the native membrane, including the currents mediated by the channels not yet molecularly identified. Osmotically activated anionic current with outward rectification (ORIC) is a dominant current in the membrane of cytoplasmic droplets under the conditions of hypoosmotic stimulation. We have previously reported remarkable functional similarities of ORIC with the vertebrate volume regulated anionic current (VRAC), such as dose-dependent activation by osmotic difference, ion selectivity sequence, and time and voltage dependent profile of the current. Using the patch clamp method on the CD membrane, we further resolve VRAC-like ORIC characteristics in this paper. We examine the inhibition by extracellular ATP and carbenoxolone, the permeation of glutamate in presence of chloride, selectivity for nitrates, and activation by GTP, and we show its single channel behavior in excised membrane. We propose that ORIC is a functional counterpart of vertebrate VRAC in filamentous fungi, possibly with a similar essential role in anion efflux during cell volume regulation.

## 1. Introduction

**Figure 1:**
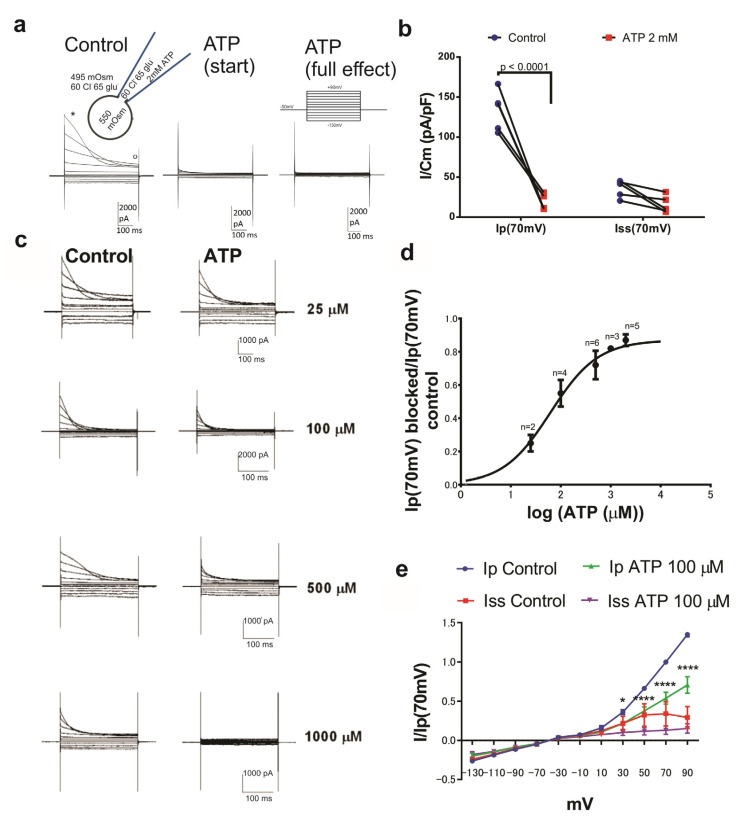
ATP blocks ORIC in a dose-dependent manner. (**a**) Representative recordings of an effect of 2 mM ATP on ORIC. The star and circle on the far left panel mark the points for measurement of peak current (Ip) and steady state current (Iss), respectively. The standard voltage protocol used to activate ORIC is depicted above far right current recording. Inset on the top left: schematic of the standard conditions of the experiment for ORIC activation, indicating the osmotic and anion content of the solutions in the pipette and bath chamber. (**b**) ORIC current densities for Ip and Iss before (Control) and after (2 mM ATP) addition, all points shown, with lines connecting the corresponding control and treatment responses of the same CD. Two way ANOVA for repeated measures with Holm–Sidac correction. (**c**) Representative recordings of ORIC inhibition by a series of concentrations of applied ATP. (**d**) ATP dose-response curve, shown as ATP-blocked fraction of Ip(70 mV) dependency on log concentration of ATP. Fit with a sigmoid dose-response curve with variable slope. (**e**) Current/voltage curves of peak and steady-state currents, normalized to control Ip value at 70 mV, from recordings of control and treatment with ATP concentration close to IC50 (100 μM). Values shown: mean ± SE. Two way ANOVA with Holm–Sidac correction for multiple comparisons., Confidence level for statistical significance was: 0.05 (*), 0.0001 (****). 30 mV: *p* = 0.0465, 50, 70 and 90 mV: *p* < 0.0001.

Cells generally react to changes in the environment using a multitude of responses that occur on different timescales. The slowest responses occur through transcriptional and genomic regulation. Moderately fast responses include activation of the cytoplasmic signal transduction pathways. The fastest responses are mediated by the plasma membrane and involve signaling through ion channels and transporters. Signaling across the plasma membrane through ion channels is the least studied class of responses in fungi, although it is certainly important for fungal physiology. Up until today, fewer than ten ion channel currents have been identified in the plasma membrane of filamentous fungi: several anionic [1,2,3] and cationic [4,5,6,7] currents and proton [8] channel currents. Among ion channels of filamentous fungi, the best-known example is the voltage-gated calcium channel Fig1 which is required for vegetative growth as well as sexual and asexual development [9]. However, other ion channels and transporters are also involved in crucial aspects of fungal physiology. For instance, it was found that more than 30% of all genes essential for the pathogenicity of *Leptosphaeria lindquistii* to sunflower plants encode transport and secretory proteins [10]. Recent studies have looked at the effects of unbalance in fungal transporter systems and found that growth cessation [11], changes in multiple drug resistance development, and virulence [12] occur—all primarily resulting from changes in K^+^, Na^+^, and proton transport [11]. Membrane transport of small organic molecules has been studied more extensively and a number of membrane transporters is identified in filamentous fungi––for organic acids, mediating uptake and secretion of carboxyl acids [13], and for amino acids, belonging to either the amino acid-polyamine-organocation (APC) [14] or the major facilitator superfamily (MFS) [15], with important physiological roles, such as in growth as well as responses to heat and cell-wall stress.

The response of filamentous fungi to osmotic stress is an interesting phenomenon for studying the involvement of ion channels, as fungi have a pressing need to mount the osmotic response quickly enough in order to adapt [16,17]. The measurements of ion fluxes during osmotic stress in *Neurospora* found net transmembrane fluxes of H^+^, K^+^, Ca^2+^, and Cl^−^ in hyphae, indicating that substantial ion current responses are present in the fungal plasma membrane [18]. Little is known about currents comprising osmotic response in fungi, except the knowledge inferred from studies in yeast [19], which are actually much simpler organisms than filamentous fungi due to their regressive evolution [20]. To explore the existence of the osmotic response machinery characteristic for yeast in filamentous fungi, a knockout of SskA homologue of a key component of *S. cerevisiae* hypoosmotic stress sensing was studied in *A. nidulans*, and it was found that it leads to only a slightly reduced capability of osmotic adaptation of *A. nidulans*, suggesting that more complex mechanisms are involved in the filamentous fungi response [21]. Notably, there are no anion channels involved in the stress response in yeast [22].

We have previously described an osmo-responsive ion current of filamentous fungi, ORIC [23]. Anionic membrane current, ORIC, in the membrane obtained from filamentous fungus *Phycomyces blakesleeanus* sporangiophore growth zone is activated by hypoosmotic swelling. The sporangiophore of *P. blakesleeanus*, characterized by fast growth responses to a variety of sensory stimuli, is a model for fungal sensory transduction [24]. As shown by us, ORIC is an outwardly rectified inactivating current that mediates chloride efflux at resting membrane potentials [23]. It is activated in a dose-dependent manner by the difference in osmotic potentials between the external and internal sides of the membrane [23]. ORIC has many characteristics that resemble volume regulated anion channel of vertebrates (VRAC): activation by osmotic swelling and selectivity for anions, increase in current conductance at more depolarized membrane potentials (the outward rectification), current inactivation at depolarized potentials, and characteristic permeability sequence (I^−^ > Cl^−^ > bicarbonate > glutamate > gluconate) [23]. Due to the pivotal role of VRAC in regulatory volume decrease during osmotic swelling of animal cells, key inhibitors of this channel as well as modulating cascades are well defined. Previous studies have demonstrated an effective block by carbenoxolone (CBX) [25] or external ATP [26]. Furthermore, VRAC is activated via internal GTP even in the absence of hypoosmotic stimulus [27]. It is involved in glutamate extrusion from cells during osmotic swelling [28] and blocked by ATP on the external side of the membrane [29]. We hypothesized that ORIC could be functionally homologous to VRAC and have additional current properties similar to those already described for VRAC.

In this work, continuing the exploration of ORIC, we show, for the first time, a number of its physiologically important properties. First, we demonstrate that extracellular ATP effectively inhibited ORIC, current in the *P. blakesleeanus* sporangiophore-derived membrane, in a dose-dependent manner. Similar to VRAC, CBX also inhibited ORIC only at positive membrane potentials. Second, we show that ORIC is inhibited by flavonoid, and could be activated by non-hydrolysable GTP binding in the absence of hypoosmotic stimulus. Third, ORIC is also carried by organic anions, namely, glutamate, and also by nitrates, indicating conduction similar to VRAC and pointing to the important role of the current in transport during osmoregulation. Finally, we utilized the knowledge of effective ORIC inhibition via ATP and demonstrated ORIC-mediating channel current in excised outside-out patches. Our findings point to the similarity of the sporangiophore-derived membrane response to that of vertebrate cells, and suggest that the anionic efflux that carries small metabolites and is activated by cell swelling in hypoosmotic conditions could be a fundamental cellular mechanism, acting in filamentous fungi as well. Understanding the physiological mechanisms of the osmotic response in filamentous fungi is essential for future advances in our understanding of fungal physiology, with potential applications in biotechnology, conservation of ecosystem biodiversity, and suppression of human and plant pathogens.

## 2. Materials and Methods

### 2.1. Model System

Wild-type strain of the fungus *Phycomyces blakesleeanus* (Burgeff) NRRL1555 (-), a saprophytic filamentous fungus form order Mucorales, was cultured on solid potato-dextrose medium by a standardized protocol [30] in glass vials, at 20–23 °C, for 72 h to allow the development of the mycelium bearing sporangiophores in a IVb stadium (with black sporangium). The mycelium is capable of producing new sporangiophores, after removal of the used ones within 24 h, up to 3 times.

### 2.2. Preparation of Cytoplasmic Droplets

In our research, we exploit the ability of the giant sporangiophores of *P. blakesleeanus* to form cytoplasmic droplets (CDs), when the growth zone is incised in an adequate solution. CDs were prepared according to a standardized protocol [23,31]. The growth zone of sporangiophores submerged in solution was incised, releasing the content under pressure. In order to increase the success rate of stable pipette–membrane contacts and enable membrane excision, CDs were immobilized during recordings by previously coating the glass bottom of the microscope chamber with concanavalin-A (100 µg/mL). CDs could be visualized immediately after mounting of the microscopic chamber. CDs were left to settle on the chamber bottom, and subsequently washed if needed, to remove the cellular debris and spores. CDs contain dense granulated cytoplasm that is always in motion, enclosed by a membrane. The membrane of CDs corresponds to plasma membrane as it has the ability to regenerate the cell wall [23].

### 2.3. Solutions and Chemicals

Standard extracellular solution (SE), used for CD formation and ORIC recording unless otherwise noted, contained (in mM): 65 KCl, 60 K-glutamate, 10 HEPES, 2 MgCl_2_, 1 CaCl_2_, adjusted to 495–505 mOsm by sucrose, and pH adjusted to 7–7.1. SE was used in experiments testing the effect of GTPγS and blocking agents. Standard pipette solution (SP), hyperosmotic with respect to extracellular solution, used for “hypoosmotic” activation of ORIC contained (mM): 65 KCl, 60 K-glutamate, 10 HEPES, 2 EGTA, adjusted by sucrose to 550 mOsm. Standard osmotic difference between SE and SP used for ORIC activation was 55 mOsm. For measurements of the effect of blockers (ATP, CBX, and Ly294002), 2 mM Na_2_ATP (Sigma Aldrich, Burlington, MA, USA) was added to SP to prevent ORIC run-down. For single channel recordings, K-glutamate was substituted with KCl in both SE and SP, so total concentration of KCl was 125 mM in both SE (125 Cl^−^ SE) and SP (125 Cl^−^ SP) solutions. For isosmotic control and GTPγS (Sigma Aldrich, Burlington, USA) experiments, SP content was modified so osmolarity adjusted with sucrose was 500 mOsm, with osmolarity identical as SE.

In experiments with varied concentrations of chloride and chloride with glutamate, SE and SP were symmetrical, with SP osmolarity adjusted to 550 mOsm with sucrose, whereas SE osmolarity was 500 mOsm. Chloride concentrations (mM) 10, 30 and 60 were supplemented with K-glutamate up to 125 mM in Cl^−^ + glutamate solutions. In Cl^−^ solutions without glutamate, chloride concentrations (mM) 10, 30, and 60 were supplemented with sucrose.

To test the conductivity of NO_3_^−^ and PO_4_^3−^, SE and SP were used in which KCl and K-glutamate were substituted with (in mM): 125 KNO_3_ and 125 KH_2_PO_4_, respectively. SE contained 6 mM Cl^−^ (from 2 MgCl_2_ and 1 CaCl_2_) and 2 mM KCl was added to SP in order to ensure that Ag/AgCl electrode would function properly. Symmetric SE and SP (55 mOsm hyperosmotic to SE) were used to test glutamate conductivity, with KCl substituted with K-glutamate. Both glutamate SE and glutamate SP contained 6 mM Cl^−^.

CBX and ATP (Sigma Aldrich, Burlington, USA) were diluted in SE in stock solutions of 2 mM and 50 mM, respectively, and diluted to a final chamber concentration by acute application to the chamber during the experiment. The synthetic quercetin analogue Ly294002 (Sigma Aldrich, Burlington, USA), non-permeable to the cell membrane, was prepared as a 10 mM stock solution in DMSO, and diluted in SP to the final concentration of 10 µM on the day of the experiment, to be applied in the final concentration through patch pipette during recording.

### 2.4. Patch Clamp Experiments

Pipettes were pulled from thick-walled borosilicate glass with filament (GB150F 0.86 × 1.50 × 100 mm, Science Products and Sutter, Novato, CA, USA) on a P97 automatic pipette puller (Sutter Instruments, Novato, USA). Pipettes used for whole cell recording had a resistance of 5–7 MΩ, to allow seal formation and break-in to the CD membrane using light suction. Pipettes used for membrane excision upon whole cell entry had 7–10 MΩ resistance to create a membrane patch of the size that would contain a sufficient number of ion channels to produce a discernible inactivation pattern while still allowing high recording gain to distinguish single channel-level transitions. Pipettes were fire-polished using a micro-forge system (L/MCPZ 101, List Medical-Elektronic) once for whole-cell recordings and twice for single-channel recordings. A microscopic chamber containing the prepared CDs was mounted on an inverted microscope (Zeiss Axiovert 10, Oberkochen, Germany) with a manual Luis & Newman micromanipulator.

For excised patch recordings, CD was first subjected to osmotic swelling by SP in the whole-cell configuration. Only after recording ORIC in such a configuration was the pipette pulled away to obtain outside-out configuration in an excised patch of the membrane. Immediately after membrane excision, depolarizing steps were applied in order to record the ORIC “steps”-like inactivation pattern.

Currents were measured using AM Systems 2400 amplifier (AM Systems, Washington, DC, USA) and Axopatch 200B (Molecular Devices, San Jose, CA, USA) and digitized at 10 kHz using Digidata 1200/1550 (Molecular Devices, San Jose, USA). For the purpose of whole-cell recordings, signals were low-pass filtered at 3 kHz, and for out-out recordings, depending on the noise level, for up to 10 kHz. Recordings were processed in Clampex 10/11.2 software (Molecular Devices, San Jose, USA).

### 2.5. Recording Protocols and Data Analysis

Upon entering the whole-cell configuration, standard voltage-clamp protocol was applied: holding voltage −50 mV, followed by a series of steps −110 mV to 90 mV, in 20 mV increments. The duration of each step was 500 ms, and the resting period in between sweeps was of 0.5−1 s. The standard protocol was used for both whole-cell and outside-out recordings.

After break-in in the whole cell configuration, with SP in the pipette, the hyperosmotic solution in the pipette dialyzed CD, inducing activation of ORIC. ORIC activation was fully achieved after one minute, as we had previously standardized [23]. ATP or CBX were applied to the bath after obtaining the control recording in the third minute of whole-cell configuration, and ORIC was recorded in the second minute after the application in order to ensure that full effect was achieved. Series resistance was not compensated. Membrane and access resistance were routinely monitored between recordings to ensure stability of whole cell recording: the minimal acceptable ratio of Rm/Ra was 5, and Ra values were not to fluctuate more that 20% through the experiment. Rm values often increased when ORIC was inhibited, as described previously [23].

Clampfit and Graphpad Prism software were used for data analysis. Current amplitude at the start and at the end of the current response to the voltage step, Ipeak (Ip) and Isteady-state (Iss), were divided by Cm to obtain current densities Ip/Cm and Iss/Cm. ORIC conductance for different anions was calculated as g = Ip/(V − V_rev_), where V_rev_ was determined from the ramp curves for the respective anion solution combination (given in the Supplementary Materials). Voltage dependent properties were measured by fitting the current-voltage curve with the Boltzman function (Equation (1)), where V_0.5_ is half voltage of the activation or inactivation, and Z_D_ is the number of gating charges.

(1) $$FIS\left(V\right)=FISmin+\frac{FISmax-FISmin}{1+e^{z_{DF(V_{0.5}-V)/RT}}}$$

Outside-out patches from smaller membrane parts obtained by pipettes above 10 MΩ could be recorded from at higher gains, which permitted a good resolution that made single channel levels measurable. The effect of extracellular ATP was measured by comparing the area under the recording (I × t), quantified in Clampfit, between the treatment and control.

To test statistical significance, different treatments were compared using two-way ANOVA for repeated measures with Holm-Sidac correction, and paired or unpaired two tailed *t* test with Welch’s correction for unequal variances. The confidence level for statistical significance was: 0.05 (*), 0.01 (**), 0.005 (***), 0.001 (****). Values obtained as parameters of the fit (IC_50_, V_50_, Z_d_) are reported as mean ± SE. In the text, all other values are reported as mean ± SD. In the graphs, reported values are mean ± SE.

## 3. Results

### 3.1. ATP Blocks ORIC

In order to explore if ORIC is sensitive to external ATP, we first tested the effect of 2 mM ATP. Osmotically-induced inactivating anion currents corresponding to ORIC were elicited by 500 ms voltage steps from a holding potential of −50 mV. ORIC recordings of control and ATP-treatment were measured at the beginning and the end of the voltage step response to extract peak current amplitude (Ip) and steady-state current amplitude (Iss), respectively (annotated in Figure 1a, first recording). As shown in Figure 1a, 2 mM ATP dramatically attenuated ORIC. Less than 1 minute after the addition of 2 mM ATP, ORIC was diminished, and after 90 s (full effect), the inactivating part of the current was completely abolished. The current density of Ip measured at +70 mV (Ip(70 mV)) was reduced (*p* < 0.0001) from 133 ± 25 pA/pF in the controls to 18 ± 10 pA/pF when 2 mM ATP was added (Figure 1b). On the other hand, the current density of Iss at +70 mV (Iss (70 mV)) was not significantly changed by the treatment: 36 ± 11 pA/pF in control and 16 ± 11 pA/pF in 2 mM ATP. We tested a range of concentrations (lower end: 25 µM) to construct a dose-response curve for the ATP effect on ORIC (Figure 1c,d). Fitting the sigmoidal dose-response curve to the blocked current fraction data ((I_control_-I_ATP_)/I_control_) showed that half inhibitory concentration (IC_50_) of ATP for ORIC was 60 µM (CI: 20–195 µM). Hill coefficient was 0.91 ± 0.3, suggesting that the ATP-inhibitory effect does not require cooperative binding of more than one ATP molecule. The current-voltage dependence of ORIC at ATP concentration close to IC_50_ (100 µM) is compared with the control in Figure 1e. Apparently, ATP effect is voltage-dependent, since ATP did not inhibit ORIC at the negative potentials. ATP affected only the inactivating part of the current (Ip), with significant inhibition starting at +30 mV potentials.

### 3.2. ORIC Inhibition by Carbenoxolone and Intracellularly Applied Membrane Impermeant Flavonoid

**Figure 2:**
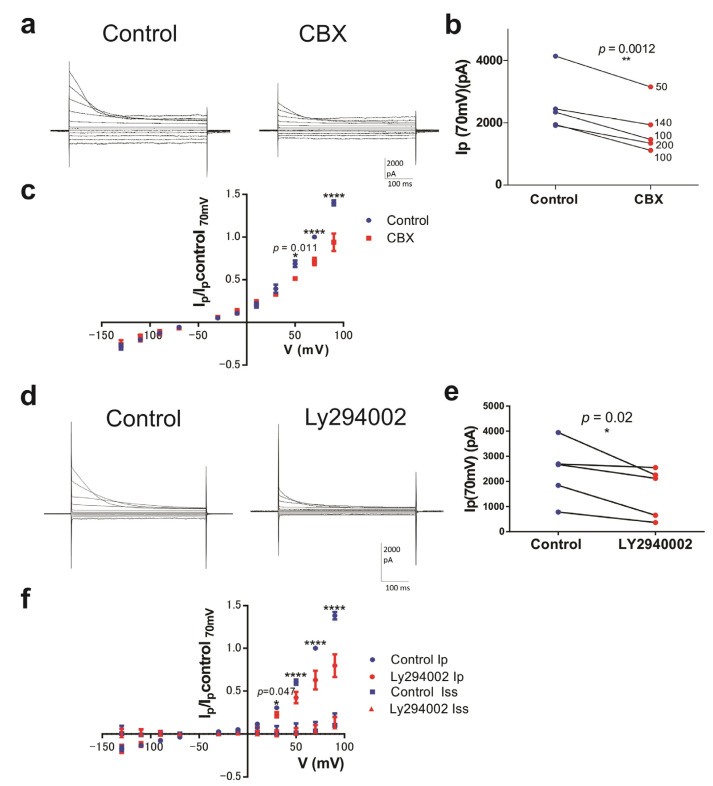
ORIC is blocked by CBX and by a membrane impermeable flavonoid. (**a**) Representative recordings of ORIC before (Control) and after CBX addition. ORIC was evoked by standard protocol, in standard conditions of the experiment for ORIC activation, as shown in Figure 1a. (**b**) Ip(70 mV) before (Control) and after (CBX) addition all points shown, with lines connecting corresponding control and treatment measurements. Concentration of CBX used is indicated next to each treatment point. Paired *t*-test, repeated measures. (**c**) IV plot of ORIC amplitude normalized to Ip(70 mV) in the control recording: before (Control) and after (CBX) addition. ANOVA with repeated measures and Holm–Sidac correction. (**d**) Representative recordings of ORIC at the beginning of recording series (Control) and after 2 min of recording with LY2940002 in pipette solution. ORIC was evoked by the standard protocol. (**e**) Ip(70 mV) Control and LY2940002 effect (after 2 min), all points shown, with lines connecting corresponding control and treatment measurements. Paired *t*-test, repeated measures. (**f**) IV plot of ORIC Ip and Iss amplitude normalized to Ip(70 mV) in control recording: Control and LY2940002 effect. ANOVA with repeated measures and Holm–Sidac correction. Confidence level for statistical significance was: 0.05 (*), 0.01 (**), 0.0001 (****). Values shown: mean ± SE.

Carbenoxolone (CBX), the blocker of pannexin channels and inhibitor of VRAC [25], was tested on ORIC. CBX had a weak inhibitory effect on ORIC (Figure 2a), as it reduced Ip(70 mV) by 27 ± 8% (n = 5).

To inspect current-voltage relationship of the CBX effect on ORIC, we plotted Ip normalized to Ip(70 mV) of the control measurement for each CD (Figure 2b), and found significant inhibition only at potentials of +50 mV or greater. The effect was also significant (*p* = 0.0012) when Ip(70 mV) before and after CBX addition were compared (Figure 2c).

To further explore the pharmacological properties of ORIC, we tested whether a flavonoid acting on the intracellular side, LY2940002, can block ORIC. The synthetic flavonoid compound LY2940002 cannot pass through the membrane, and since we applied it through the pipette, the ORIC-blocking effect would confirm that the flavonoid binding site mediating its effect on ORIC is on the internal side of the membrane. LY2940002 inhibited ORIC significantly (Figure 2d–f): at 70 mV, inactivating current (Ip-Iss) was inhibited by 37 ± 20%. Comparison of normalized current values showed that inhibition was significant at depolarized potentials (+50, +70 and +90 mV), confirming that ORIC inactivating current is sensitive to inhibition by flavonoid LY2940002 on the internal side of the membrane.

### 3.3. Glutamate Can Be Carried by ORIC

**Figure 3:**
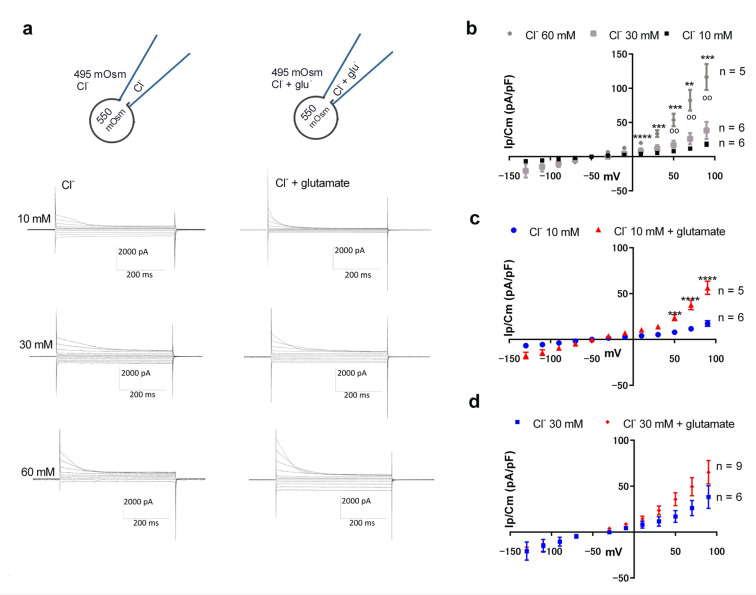
ORIC is carried by glutamate under low chloride conditions. (**a**) Representative current recordings obtained with symmetrical solutions with varied anion concentrations. Insets on the top: schematic of the conditions of the experiment for ORIC activation, indicating the osmotic and general anion content of the solutions in the pipette and bath chamber, with exact anion concentrations given for each row of current recordings. Top row: 10 mM Cl^−^ (left), 10 mM Cl^−^ with 115 mM glutamate (right). Middle row: 30 mM Cl^−^ (left), 30 mM Cl^−^ with 95 mM glutamate (right). Bottom row: 60 mM Cl^−^ (left), 60 mM Cl^−^ with 65 mM glutamate (right). (**b**) Voltage dependency of ORIC peak current density in 10 mM, 30 mM, and 60 mM Cl^−^ solutions without glutamate. Statistical difference (two-way ANOVA, Holm-Sidac correction) is marked with * for comparison to 10 mM Cl^−^ series and with ^o^ for comparison to 30 mM Cl^−^ series. (**c**) Voltage dependency of ORIC peak current density in solutions containing 10 mM Cl^-^ and 10 mM Cl^-^ with 115 mM glutamate series plotted for comparison. Two way ANOVA with Holm–Sidac correction. (**d**) Voltage dependency of ORIC peak current density in solutions containing 30 mM Cl^−^ and 30 mM Cl^−^ with 95 mM glutamate series plotted for comparison. In all recordings, the standard voltage protocol was applied as shown in Figure 1. Confidence level for statistical significance was: 0.01 (**), 0.005 (***), 0.0001 (****).

We have previously shown that ORIC-mediating channels weakly permeate glutamate [23] (P_glut_/P_Cl_ = 0.09) when these anions are examined in separate experiments. Since the interior of the fungal cell is expected to always contain some chloride concentration, it would be important to test if ORIC permeates glutamate under similar conditions. We first measured ORIC currents in symmetrical solutions containing varied Cl^−^ concentrations (10, 30, and 60 mM) (Figure 3a left and Figure 3b), and found that the increase in the Cl^−^ concentration from 10 to 30 mM had no significant effect on ORIC density. In 60 mM Cl^−^, ORIC was significantly larger at depolarized potentials (+50 mV and above) than in both 30 mM and 10 mM Cl^−^ (Figure 3b). Then, we measured ORIC in symmetrical solutions with the same varied Cl^−^ concentrations, supplemented with glutamate to the total of 125 mM. For comparison, ORIC in 125 mM glutamate is given in Supplementary Figure S2a,b, to show that the increase in ionic strength alone (as in the Supplementary Materials Figure S2b from 10 mM Cl^−^ to 125 mM glutamate) is not sufficient to elicit an increase in ORIC. We have previously shown ORIC dependency on Cl^−^ concentrations when it is combined with glutamate (supplemented to 125 mM), and here we compare it with dependency on Cl^−^ concentration when glutamate was not present.

When a low Cl^−^ concentration (10 mM) was combined with glutamate (Figure 3c), current density at all depolarized potentials was significantly higher (*p* = 0.0034) (Ip/Cm(70 mV) =37.6 ± 15 pA/pF) than with the same concentration of Cl^−^ without glutamate (Ip/Cm(70 mV) = 11.7 ± 3.7 pA/pF). It should be noted that in the solution containing only glutamate (125 mM), current density is smaller at +70 mV (*p* = 0.001) and at other depolarized potentials (Ip/Cm(70 mV) = 10.5 ± 4.3 pA/pF) than in 10 mM Cl^–^ with 115 mM glutamate (Supplementary Materials Figure S2b), demonstrating that ORIC carries glutamate better when it is combined with Cl^−^, at low concentration. For 30 mM Cl^−^, ORIC density was similar with and without glutamate (Figure 3d), as it was for 60 mM Cl^−^, with Ip/Cm(70 mV) = 82 ± 30 pA/pF (n = 4) in 60 mM Cl^−^ and Ip/Cm(70 mV) = 79 ± 55 (n = 14) in 60 mM Cl^−^ with glutamate (65 mM).

### 3.4. ORIC Permeates Nitrate as well as Chloride

**Figure 4:**
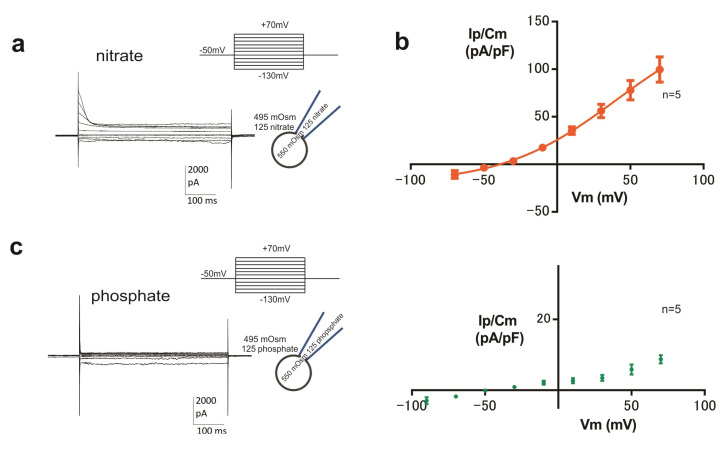
Nitrate ions are carried by ORIC, while phosphate ions are not. (**a**) Representative recording of ORIC evoked with voltage protocol shown in symmetrical 125 mM NO^−^_3_ solutions. Inset on the right: schematic of the conditions of the experiment for ORIC activation, indicating the osmotic and anion content of the solutions in the pipette and bath chamber. (**b**) IV plot of ORIC peak current density in symmetrical 125 mM NO_3_^−^ solutions. (**c**) Left: Representative recording of ORIC evoked with a voltage protocol shown in symmetrical 125 mM phosphate solutions. Inset: schematic of the conditions of the experiment for ORIC activation, indicating the osmotic and anion content of the solutions in the pipette and bath chamber. Right: IV plot of ORIC peak current density in symmetrical 125 mM phosphate solutions.

Next, we explored whether ORIC, as the dominant osmotically activated anion current in the CD membrane, can be carried by nitrate and phosphate ions. Using 125 mM NO_3_^−^ (nitrate) solutions and with a standard osmotic difference of 55 mOsm, a prominent ORIC was recorded (Figure 4a). The voltage dependency of current density in nitrate solutions (Figure 4b) was similar to ORIC with SE and SP based on chloride. Half activation voltage obtained from the Boltzmann fit of IV curve obtained from recordings in nitrate solutions was V_50_ = 40 ± 3 mV, and the number of gating charges was approximately 1 (z_d_ = 0.98 ±0.12), showing that ORIC is still a moderately rectified current when carried by nitrates. Regarding the size of the current, in nitrate solutions Ip/Cm(70 mV) was 100 ± 30 pA/pF (n = 5), which was not different than in 125 mM Cl^−^ (129 ± 64 pA/pF, n = 22) measured previously [23]. This result confirms that nitrate is equally efficient as chloride in permeating through the channel that mediates ORIC, and that ORIC could serve as an efflux pathway for nitrates at resting membrane potentials. We also tested solutions containing phosphate anions (Figure 4c). It can be seen that phosphate does not permeate well through channels mediating ORIC, and the outwardly rectified and inactivating current could not be recorded in phosphate solutions. (Note that the different scaling of the y-axis in the IV graph of ORIC density in phosphate solutions.)

### 3.5. GTP Analogue Activates Current with Properties of ORIC, without Osmotic Stimulation

**Figure 5:**
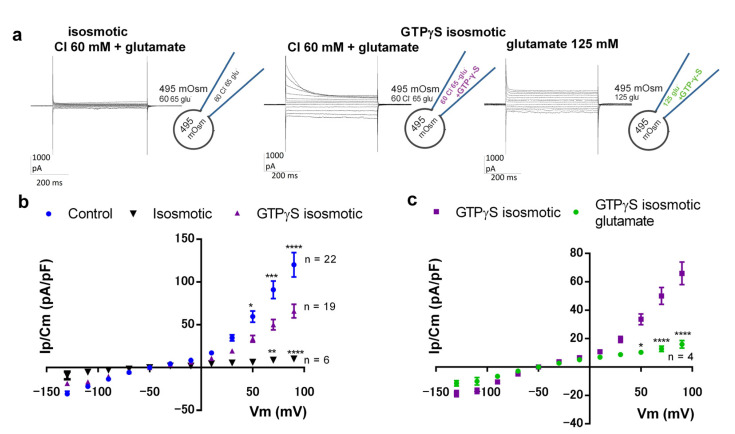
GTP analogue, in the absence of osmotic stimulus, activates prominent outwardly rectified anionic current that resembles ORIC. (**a**) Representative current recordings, evoked with standard voltage stimulation protocol as depicted in Figure 1a, without osmotic difference between bath and pipette solution. Insets on the right of each current recording: schematic of the conditions of the experiment for ORIC activation, indicating the osmotic and anion content of the solutions in the pipette and bath chamber. Left to right: isosmotic Cl^−^ -based solutions; GTPγS in pipette, with isosmotic Cl^−^-based solutions; GTPγS in pipette, with isosmotic glutamate -based solutions. (**b**) Voltage dependence of current densities obtained for ORIC recorded in “hypoosmotic” Cl^−^-based solutions (Control group), isosmotic Cl^−^-based solutions (Isosmotic group) and GTPγS-induced current in isosmotic Cl^−^-based solutions (GTPγS isosmotic). (**c**) IV curve of GTPγS-induced current in symmetrical glutamate solutions. Curve for isosmotic GTPγS-induced current in Cl^−^-based solutions, shown for comparison. (**b**,**c**) Values shown are mean ± SE. Two way ANOVA with Holm–Sidac correction for multiple comparisons. Confidence level for statistical significance was: 0.05 (*), 0.01 (**), 0.005 (***), 0.0001 (****).

The current recorded in isosmotic symmetrical solutions with 60 mM Cl^−^ (at +70 mV) was of very small density, 9 ± 3 pA/pF (n = 4) and devoid of inactivating current at depolarizing potentials, which is characteristic for ORIC (Figure 5a left). Upon inclusion of 125 µM GTPγS to the pipette solution, the same standard voltage stimulation protocol evoked the current family resembling ORIC, with a large inactivating current at depolarizing potentials (Figure 5a middle). GTPγS-activated Ip(70 mV) was 50 ± 6 pA/pF (n = 19), significantly larger than the current under the same conditions without GTPγS (*p* = 0.0045 at +50 mV, and *p* < 0.0001 at +70 mV and +90 mV). Osmotically activated ORIC, with the same Cl^−^ concentration, had Ip = 91 ± 10 (n = 22) (see Figure 5b), which was larger than the GTPγS-activated current. Half activation voltage obtained from the Boltzmann fit of the IV curve with GTPγS was V_50_ = 52 ± 2 mV and the number of gating charges was approximately 1 (z_d_ = 1.3 ± 0.1), demonstrating that the GTPγS-induced current is moderately rectified. Those parameters obtained from Boltzmann fit for GTPγS-induced current were indistinguishable from the values obtained for the control ORIC (with the same solutions, but with 55 mOsm hyperosmotic solution in the pipette, without GTPγS) (Figure 5b): V_50_ = 50 ± 2 mV and z_d_ = 1.4 ± 0.2. Substituting all Cl^−^ for glutamate reduced GTPγS-activated current (Figure 5a, right and Figure 5c). In glutamate, Ip(70 mV) was 13 ± 2 pA/pF (n = 4), significantly reduced from GTPγS-activated Ip(70 mV) with Cl^−^, demonstrating that GTPγS-activated current is mediated by anions, as is ORIC. To further confirm that GTPγS-activated current indeed shares biophysical properties with ORIC, we compared the voltage dependency of the fraction of inactivating current (FIC = (Ip − Iss)/Ip) (Supplementary Materials Figure S1a) and the speed of depolarization-induced current inactivation measured as τ_in_ (Supplementary Materials Figure S1b) with the same properties of ORIC, and we found that the GTPγS-activated current and ORIC cannot be distinguished based on these properties.

### 3.6. ORIC Can Be Recorded in Excised Patches

**Figure 6:**
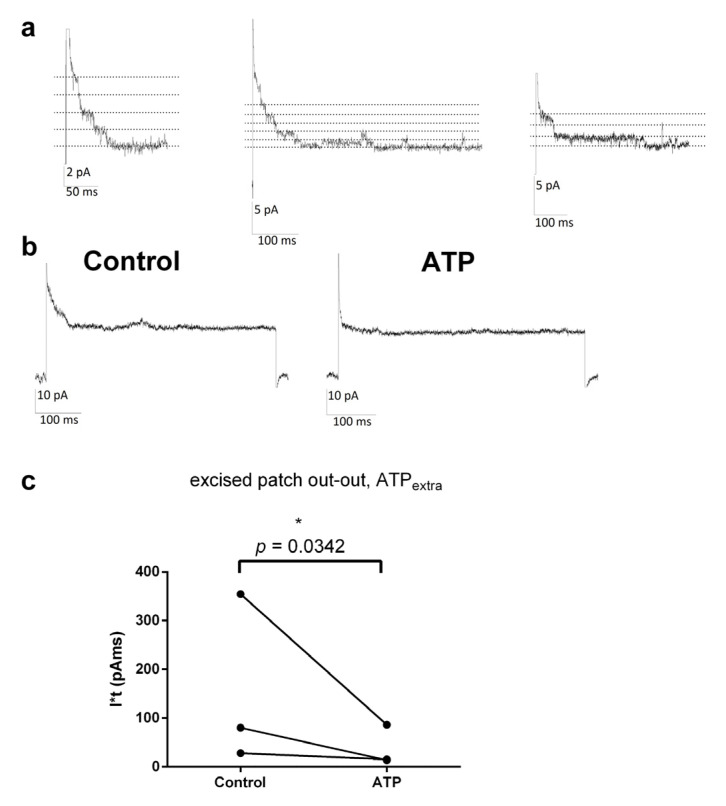
Single channel current corresponding to ORIC can be recorded in excised patches in out-out configuration. (**a**) Representative recordings from three different excised patches, of currents evoked by a step to +70 mV from −50 mV holding potential, as in the ORIC voltage stimulation protocol. Dotted lines mark the open channel current levels and scale bars are given for each recording. (**b**) ATP applied on the external side of the membrane blocks current evoked by ORIC stimulation protocol. Representative excised patch current recordings before (Control) and after (ATP) addition of 2 mM ATP to bath solution. (**c**) Graph shows area (I*t) under the inactivating part of the current, quantified for Control and ATP, in three independent excised patches. Paired *t*-test, with lines connecting paired measurements. Confidence level for statistical significance was: 0.05 (*).

Finally, the properties of ORIC in excised patches were examined. ORIC was first evoked and recorded in the whole-cell configuration, using “hypoosmotic” Cl^−^-based solutions. Immediately after obtaining the excised membrane patch in the outside-out configuration, the depolarization stimulus was employed. The obtained currents (Figure 6a), activated by depolarization to +70 mV, inactivated in less than 50 ms, giving the characteristic appearance of “steps” with a few apparent re-openings during the inactivation period. The amplitude of the single channel current was estimated from the transitions in four independent patches at +90 mV (mean ± SD) = 1.2 ± 0.3 pA, and at +70 mV (mean ± SD) = 0.9 ± 0.1. At lower depolarizations, the transitions could not be discerned reliably. From obtained single channel amplitude, at depolarized potentials, single-channel conductance was approximately 15 pS in symmetrical 125 mM KCl solutions. The evoked inactivating current could be activated again after allowing the rest period at hyperpolarized potential (Supplementary Materials Figure S3). To confirm that recorded “steps”-like current in excised patches has characteristics of ORIC, we tested if it can be inhibited by 2 mM ATP applied to the bath side of the membrane (Figure 6b). We used the area under the inactivating part of the current as a measure of current activity instead of simply current amplitude in order to account for eventual non-synchronous current activity in the observed period. As can be seen from the graph in Figure 6b, ATP treatment significantly reduced the inactivating current area (*p* = 0.034), suggesting that the inactivating current recorded in the excised patches, in the conditions that activate ORIC in the whole cell configuration, is the same as ORIC.

## 4. Discussion

This work shows that the osmotically activated current ORIC in the membrane from the sporangiophore of filamentous fungus *Phycomyces blakesleeanus* has several newly identified properties of interest for fungal physiology. The potent inhibition by ATP characterizes ORIC as an external ATP-sensing current, which to the best of our knowledge, is the only element of the membrane signaling with such an ability identified in the filamentous fungi membrane, while the finding that glutamate can be carried by ORIC indicates that glutamate efflux may be involved in signaling and mediated by ion channels in fungi as well. Both of these features, along with the activation by GTPγS without osmotic stimulus, and the inhibition by carbenoxolone, are the properties that ORIC shares with the well-characterized vertebrate volume-regulated anionic current (VRAC) [32].

For fungi, there would be obvious advantages to sensing extracellular ATP as a signal released from their own damaged cells or from other organisms. Such sensing ability has been described in *Trichoderma atroviride* [33], where ATP serves as a stress signal about self-damage inducing subsequent conidiation, and in *Shiraia* sp., which release ATP in response to close interaction with co-cultured bacteria and respond to extracellular ATP with conidiation and synthesis of secondary metabolites [34]. It remains to be seen if an ORIC-like current is involved in these phenomena.

The ability of the ORIC-mediating channel to participate in the release of glutamate under conditions of low chloride concentrations and osmotic swelling shows that filamentous fungi can use membrane ion channels in small organic molecule signaling during the osmotic response, a feature usually not attributed to this group. The only other fungal channel with a similar role reported to mediate the entry of uncharged acetic acid is the membrane channel involved in solute efflux during osmoadaptation [19], aquaglyceroporin Fps1 in yeast. Aquaglyceroporins, which permeate small uncharged solutes and consequentially do not mediate ion currents, are expressed in the membrane of filamentous fungi [35]. Recently, seven homologues of the aquaglyceroporins have been identified in Aspergillus [36], but transport of small solutes has not been measured, except for the channel involvement in the hydrogen peroxide efflux.

As we have described previously [23], and again demonstrated here, ORIC shares the biophysical characteristics with vertebrate volume-regulated current. With the additional VRAC-like properties of ORIC characterized here, it is clear that ORIC is a physiological counterpart of VRAC, the current mediated by the channel that is not found outside the chordate group. There is no other ion current, besides VRAC, described in Fungi or any other kingdom of life with characteristics similar to the ones typical for ORIC. Vertebrate VRAC is ubiquitously present in vertebrate cells with the primary physiological role in the regulation of cell volume during osmotic swelling [37]. VRAC is an osmotically activated anionic current with moderate outward rectification, prominent voltage dependent inactivation at depolarized potentials, and an Einsmann type I anion selectivity sequence [38]. The protein complex forming the VRAC-mediating channel has been shown to be a hexamer composed of leucine-rich repeats containing 8A protein (LRRC8A) as the obligatory subunit [39] and various combinations of other subunits (LRRC8B-E), and its structure has been characterized in several subunit combinations [40]. VRAC channel complexes containing LRRC8E are mediators of glutamate release during edemic swelling in astrocytes [28], possibly similar to ORIC ability.

ORIC shares with VRAC such an extensive list of key current properties that it is reasonable to assume that they are serving similar cellular roles. Both currents are activated by swelling and GTP [27], and mediate efflux of anions with the same unique current “fingerprint”. We propose that ORIC represents a filamentous fungi “functional homologue” of VRAC. The fact that *P. blakesleeanus* genome does not contain an ORF sequence with any substantial homology to LRRC8A-E, leads us to the conclusion that ORIC-mediating channel might not bear extensive structural similarity to is vertebrate counterpart.

In this context, it is unexpected to find the inhibitory effect of carbenoxolone on ORIC, however weak, because this finding extends parallel with VRAC [25], suggesting that the ORIC-mediating channel probably shares some limited structural similarity with the pannexine-LRRC8(A–E) family. On the other hand, ORIC is not inhibited by 4-[(2-butyl-6,7-dichloro-2-cyclopentyl-2,3-dihydro-1-oxo-1*H*-inden-5-yl)oxy] butanoic acid (DCPIB)(unpublished data), in contrast to VRAC [41], although it is inhibited by DIDS, niflumic acid, and antracene [42], relatively nonselective anion channel inhibitors. The DCPIB-binding site in VRAC-mediating channel is the same small region that confers the inhibiting ability to ATP [26]. It is possible that some other part of the channel structure is not accommodating DCPIB binding in the case of ORIC, whereas the ATP binding site, as it has physiological importance, is conserved between VRAC and ORIC-mediating channels.

Flavonoids inhibit VRAC [43], but whether they act on the intracellular side, as we have found for ORIC, has not yet been explored. However, a recent study of the flavonoid effect on volume regulation in rat thymocytes found that among a large group of tested, externally added flavonoids, only hydrophobic ones were effective [44], and, therefore, it is possible that they act on the intracellular side on VRAC as well.

In addition to ORIC and VRAC, there are other anionic currents, mediated by bestrophins and anoctamins, that share the characteristic permeability sequence characterized by greater selectivity for iodide than for chloride ions [45], in contrast to anionic currents mediated by CLC and CFTR, whose selectivity sequence is different [46]. The selectivity sequence found in VRAC and ORIC is considered to be a hallmark of the low affinity binding sites [47], inferring that such channels might be permeable for larger anions, as shown for VRAC with glutamate, taurine, and other molecules [29], and, here, for ORIC with glutamate.

It should be noted that for VRAC, the ATP-blocking effect is considered to be a consequence of the ATP-releasing function of VRAC-mediating channels [48]. If a similar mechanism of block were shown to be correct for ORIC-mediating channels, they would serve as ATP-releasing channels, whereas the ATP sensor in fungi would yet need to be identified.

However, in this work, we found several differences between ORIC and VRAC regarding ion channel properties. First, we have shown that the ORIC-mediating channel does not permeate NO_3_^−^ much better than Cl^−^, but with roughly the same permeability. For VRAC, the permeability for NO_3_^−^ > Cl^−^ [29]. Second, the single channel conductance of ORIC at depolarized potentials (around 15 pS) is much smaller than typical for VRAC [49] at the same potentials (50–80 pS). Finally, as we reported previously, depolarization-induced current inactivation in ORIC is faster for more permeating anions [23], whereas the opposite is found for VRAC [50]. All these discrepancies corroborate the conclusion that ORIC is not mediated by LRRC8(A–E)-like proteins but by a protein substantially dissimilar, possibly due to phylogenetic distance, to the channel mediating VRAC.

Osmotic activation accomplished by hyperosmotic pipette solution, as used in our experiments and in classical VRAC studies [51], is consistent with the activation of lysine deficient protein kinases (WNKs), ancient cytoplasmic regulatory scaffolds sensitive to low [Cl^−^] and hyperosmotic cytoplasmic conditions [52]. For WNKs to be able to orchestrate regulatory volume increase in mammalian cells, there is an absolute requirement for functional VRAC to be present [53]. According to the current model in mammalian cells, cytoplasmic hyperosmotic conditions activate VRAC, enabling the efflux of Cl^−^, and thus lowering Cl^−^ concentration, providing the conditions necessary for WNK activation. There are three sequences in the *P. blakesleeanus* genome that correspond to WNKs [54]. It is plausible that, since ORIC is serving the same function as VRAC in mammalian cells (it is activated by hyperosmotic cytoplasm and it can mediate Cl^−^ efflux), it has a role as a Cl^−^ efflux channel for the activation of WNK. In *Neurospora crassa*, the ion flux changes were measured during the fludioxonil challenge that mimicked hyperosmotic cascade activation, and the authors found chloride uptake during prolonged periods of turgor recovery. Ion flux changes during the actual response of *N. crassa* to osmotic stress conditions were shown previously [18] to rely on several ion flux changes that include Cl^−^ efflux, consistent with the proposed role of ORIC.

From the recent essay dealing with the time-series of metabolomic data, it was concluded that yeasts extrude selected small metabolites to the external medium in response to stimuli from the environment, independent of possible metabolic overproduction of metabolites (“overflow”) [55]. Interestingly, in the protozoa Trypanosoma, the seemingly identical process to the CD swelling response has been described: a hypoosmotic swelling-induced volume regulatory efflux of anions accompanied by a loss of electroneutral and anionic amino acids, such as glutamate [56]. Even in bacteria, a similar mechanism of choline efflux in response to osmotic stress has been found [57].

Anionic efflux, carrying small metabolites, that is activated by cell swelling under hypoosmotic conditions and involved in regulatory volume decrease, could be a fundamental cellular mechanism, although it possibly acquired additional roles in the walled turgescent cells of filamentous fungi. Since ORIC is a dominant current in cytoplasmic droplets, the membrane model system originating from the growing part of sporangiophore, it is possible that ORIC-mediated anion efflux is involved in the growth process of the sporangiophore or reactions to various stimuli that sporangiophore can detect.

In conclusion, ORIC, swelling activated anionic current that mediates efflux of glutamate and is inhibited by extracellular ATP, is a functional counterpart of vertebrate VRAC, with the proposed roles in volume regulation, signaling, and cell growth.

## Data Availability

The data is available upon a reasonable request to the corresponding author.

## Supplementary Materials

The following supporting information can be downloaded at: https://www.mdpi.com/article/10.3390/jof9060637/s1, Figure S1. Comparison of biophysical properties of ORIC, registered with 60 Cl symmetrical solutions with hypertonic (+55 mOsm) pipette, with the properties of GTPγS-induced current registered with the isosmotic symmetrical 60 Cl. (a). Fraction of inactivating current FIC = (Ip − Iss)/Ip for GTPγS-induced current overlaps with FIC for control ORIC. Extra sum of squares comparison of Boltzmann fits found that both series of data can be fitted with the same curve. Best fit parameters for GTPγS-induced current were: half-inactivation V_50_ = 20 ± 2 mV, and the slope (RT/zF) = 14 ± 2, and for ORIC were V_50_ = 22 ± 2 and RT/z_d_ F = 15 ± 2. corresponding to the gating charge z_d_(GTPγS) = 1.85 and z_d_(ORIC) = 1.73. (b). Inactivation speed of current evoked by depolarizing pulse, expressed as the time constant of inactivation, τ_in_, has the same voltage dependency in control ORIC and in GTPγS-induced current. Extra sum of squares comparison of exponential fits found that both series of data can be fitted with the same curve. Best fit of the rate of change with voltage for GTPγS-induced current was k= 0.03 ± 0.03 mV^−1^, and for ORIC k = 0.02 ± 0.02 mV^−1^. Figure S2. Conductance properties of ORIC. (a) ORIC specific conductance in 125 mM chloride, 125 mM glutamate and 60 mM chloride with 65 mM glutamate. The bath and pipette solutions were symmetrical, except that the pipette solution contained additional 6 mM Cl^−^ (in MgCl_2_ and CaCl_2_). The conductance, calculated as g = I/(V − V_rev_), was normalized to capacitance to obtain specific conductance, shown in graph bellow. Specific conductance reflects the unitary conductance of the ion channel and the probability that the channel is opened at each potential. Specific conductance with 125 mM glutamate is very low at depolarized potentials, while for both chloride concentrations shown it rises with depolarization. ORIC in 60 mM chloride (combined with glutamate 65 mM) has roughly half of the specific conductance of ORIC in 125 mM chloride solution, suggesting that with chloride concentration 60 mM or more, glutamate contribution to the current is very small. In the graph, statistical differences (two way ANOVA, Holm-Sidac correction) are marked with * for comparison to 125 mM Cl^−^ , and with ^+^ for comparison to 125 mM glutamate. Mean ± SE, for values extracted from Ip. (b) Voltage dependence of ORIC current density in 125 mM glutamate (n = 5), with Ip/Cm in 10 mM Cl^−^ shown for comparison. Mean ± SE. Confidence level for statistical significance was: 0.05 (*), 0.005 (***), 0.0001 (****). Figure S3. Inactivating current in excised patches recovers from inactivation. The current recovery is incomplete if during recovery time of several hundred ms, depolarized potential is applied (upper row). Hyper polarized holding potential (Vh) during recovery time allows for complete current recovery (bottom row). Currents were evoked by a step to + 70 mV, and after varied time or Vh of recovery, current was evoked by the same step again. Table S1. V_rev_ of ORIC, used for extracting conductance values g = I/(V − V_rev_).
