# Supplementary material for: Osmotically Activated Anion Current of Phycomyces Blakesleeanus—Filamentous Fungi Counterpart to Vertebrate Volume Regulated Anion Current
Source: J Fungi (Basel). 2023 May 31;9(6):637. doi: 10.3390/jof9060637 (PMC10301571; doi:10.3390/jof9060637)
Supplement: Supplementary file 1 [file jof-09-00637-s001.zip › jof-2414229-Supplementary Materials.pdf]

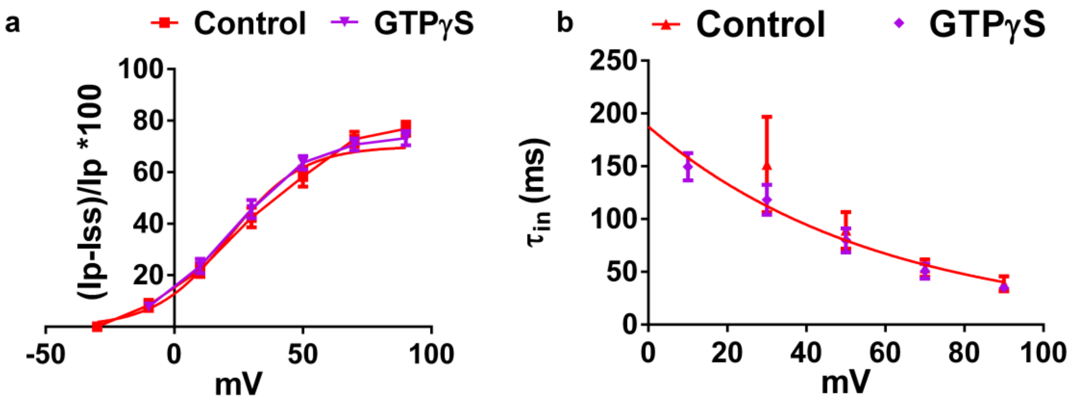

Figure S1. Comparison of biophysical properties of ORIC, registered with 60 Cl symmetrical solutions with hypertonic (+55 mOsm) pipette, with the properties of GTPγS-induced current registered with the isosmotic symmetrical 60 Cl. (**a**). Fraction of inactivating current FIC = (Ip − Iss)/Ip for GTPγS-induced current overlaps with FIC for control ORIC. Extra sum of squares comparison of Boltzmann fits found that both series of data can be fitted with the same curve. Best fit parameters for GTPγS-induced current were: half-inactivation V_{50} = 20 ± 2 mV, and the slope (RT/zF) = 14 ± 2, and for ORIC were V_{50} = 22 ± 2 and RT/z_d F = 15 ± 2., corresponding to the gating charge z_d(GTPγS) = 1.85 and z_d(ORIC) = 1.73. (**b**). Inactivation speed of current evoked by depolarizing pulse, expressed as the time constant of inactivation, τ_{in}, has the same voltage dependency in control ORIC and in GTPγS-induced current. Extra sum of squares comparison of exponential fits found that both series of data can be fitted with the same curve. Best fit of the rate of change with voltage for GTPγS-induced current was k= 0.03 ± 0.03 mV^{-1}, and for ORIC k = 0.02 ± 0.02 mV^{-1}.

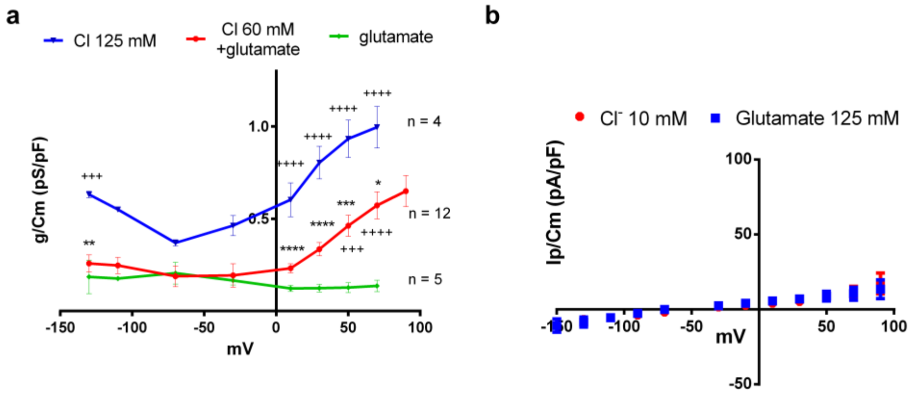

Figure S2. Conductance properties of ORIC. (**a**) ORIC specific conductance in 125 mM chloride, 125 mM glutamate and 60 mM chloride with 65 mM glutamate. The bath and pipette solutions were symmetrical, except that the pipette solution contained additional 6 mM Cl^- (in $MgCl_2$ and $CaCl_2$). The conductance, calculated as g = I/(V-V_{rev}), was normalized to capacitance to obtain specific conductance, shown in graph bellow. Specific conductance reflects the unitary conductance of the ion channel and the probability that the channel is opened at each potential. Specific conductance with 125 mM glutamate is very low at depolarized potentials, while for both chloride concentrations shown it rises with depolarization. ORIC in 60 mM chloride (combined with glutamate 65 mM) has roughly half of the specific conductance of ORIC in 125 mM chloride solution, suggesting that with chloride concentration 60 mM or more, glutamate contribution to the current is very small. In the graph, statistical differences (two way ANOVA, Holm-Sidac correction) are marked with * for comparison to 125 mM Cl^- , and with $^+$ for comparison to 125 mM glutamate. Mean ± SE, for values extracted from Ip. (**b**) Voltage dependence of ORIC current density in 125 mM glutamate (n=5), with Ip/Cm in 10 mM Cl^- shown for comparison. Mean ± SE. Confidence level for statistical significance was: 0.05 (*), 0.005 (***), 0.0001 (****).

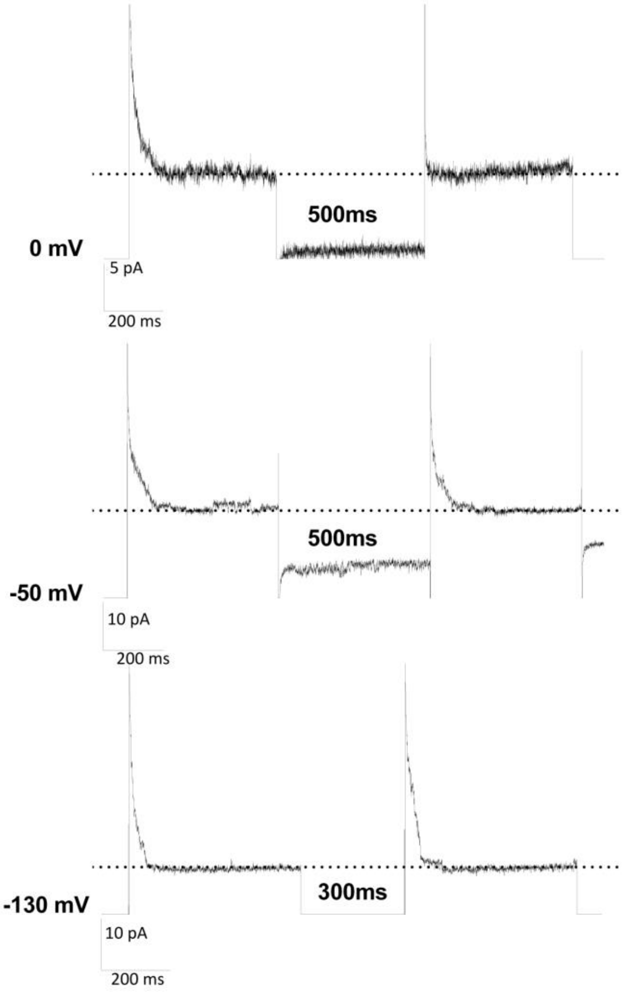

Figure S3. Inactivating current in excised patches recovers from inactivation. The current recovery is incomplete if during recovery time of several hundred ms, depolarized potential is applied (upper row). Hyper polarized holding potential (Vh) during recovery time allows for complete current recovery (bottom row). Currents were evoked by a step to + 70 mV, and after varied time or Vh of recovery, current was evoked by the same step again.

Table S1. V_{rev} of ORIC, used for extracting conductance values $g = I/(V - V_{rev})$.

V_{rev} (mV)	Cl^- 125 mM	Cl^- 60 mM +glutamate	Glutamate 125 mM
mean	-15.6	44.5	-91.9
SD	3.6	19.1	21.4
n	6	6	5
